# Tube Stoma for the Management of Ileocolic Anastomotic Leak in a Patient With Metastatic Colon Cancer

**DOI:** 10.7759/cureus.52314

**Published:** 2024-01-15

**Authors:** Mohammed I Elsayed

**Affiliations:** 1 General Surgery, Stepping Hill Hospital, Manchester, GBR

**Keywords:** abdominal sepsis, tube ileostomy, colorectal anastomotic leak, right hemicolectomy, cytoreductive surgery and hipec, invasive colon cancer

## Abstract

This case involves a 53-year-old male who was diagnosed with stenotic ascending colon cancer and peritoneal metastatic deposits. He was initially planned for cytoreductive surgery and heated intraperitoneal chemotherapy (CRS and HIPEC), along with resection of the primary tumor in the form of right hemicolectomy. Intraoperatively, the disease was found to be more extensive than anticipated. Consequently, the plan was modified to include debulking right hemicolectomy with hand-sewn ileocolic anastomosis and extensive peritoneal procedures. Postoperatively, he experienced an anastomotic leak, leading to another laparotomy. However, due to anatomical challenges, creating a stoma was considered unsafe. Therefore, innovative interventions were performed, including controlling the anastomotic defect with a 30Fr Foley catheter without disrupting the anastomosis. A collaborative effort from various medical teams facilitated the patient's discharge home after an extended stay in the critical care unit (CCU).

## Introduction

Anastomotic leak following colorectal cancer surgery is a serious complication associated with increased morbidity, mortality, and the delayed commencement of planned adjuvant chemoradiotherapy [1]. The incidence of anastomotic leak ranges from 2.8% to 30%, with 75% occurring in rectal surgeries, resulting in morbidity rates of 20-35% and mortality rates between 2% and 16.4% [2,3]. This case report is unique as no similar case has been published before, highlighting the crucial role of the multidisciplinary team in managing this morbid complication. Two notable aspects include the decision to maintain the anastomosis while inserting a Foley catheter through the defect to serve as a tube stoma. Additionally, the case emphasizes the benefits of utilizing local resources and fostering collaboration among different medical teams, such as critical care, radiology, dietitians, wound care, physiotherapy, speech and language therapy assessment, and psycho-oncology teams. The report details the case of a 53-year-old male with stenotic ascending colon adenocarcinoma and multiple peritoneal deposits who experienced an anastomotic leak following elective debulking right hemicolectomy, providing insights from both evidence-based and clinical perspectives.

## Case presentation

A 53-year-old obese male presented with a moderately differentiated adenocarcinoma of the stenotic ascending colon, multiple omental deposits, and a rectal polyp (T3 N1b M1c). At the tertiary colorectal and peritoneal multidisciplinary team (MDT) meeting, the consensus leaned towards cytoreductive surgery with right hemicolectomy and heated intraperitoneal chemotherapy, given the nature of the T3 tumor, absence of nodal involvement, and presence of omental disease. Physically, the patient had compromised mobility due to extensive surgeries on his right lower limb, suffered from sleep apnea, and regularly used a continuous positive airway pressure (CPAP) machine at home. His habits included the daily smoking of over 20 cigarettes and cannabis use.

Primary surgery

Intra-operatively, the extent of the disease exceeded expectations, including a large ascending colon tumor, multiple adherent loops of the terminal ileum to the cecum with an impression of mesenteric mass, large omental nodules encroaching on the mid-transverse colon serosa, and multiple peritoneal deposits throughout the peritoneal cavity and on the dome of right hemidiaphragm. The Peritoneal Cancer Index (PCI) was 28 [4]. Due to the disease extent, obesity, narrow pelvis, and positioning challenges, a discussion led to the decision for debulking surgery only, excluding heated intraperitoneal chemotherapy (HIPEC) due to questionable benefits in his case and cardiac risks. Moreover, as the tumor was still resectable and, due to his body habitus, an end ileostomy was not a viable option. Therefore, he had a debulking right hemicolectomy with hand-sewn ileocolic anastomosis, greater omentectomy, and peritonectomies. The Complete Cytoreduction score was 2 (CC2) [4]. The patient spent one day in the critical care unit (CCU) before transferring to the ward.

Investigations

On postoperative day (POD) 5, the patient experienced agitation, abdominal pain, nausea, and vomiting. Blood investigations were normal, and C-reactive protein (CRP) was 147 (Table 1).

**Table 1:** Laboratory investigations at baseline, and on postoperative days 5, 7, and 14.

Parameters	Reference Range	Preoperative (Baseline)	Postoperative Day 5	Postoperative Day 7	Postoperative Day 14
Hemoglobin	130–180 g/L	Within Range	Stable	114	131
White Cell Count (WCC)	4.0-11.0 x 10^9/L	Within Range	Stable	11.2	10.5
Platelet Count	150-450 x 10^9/L	Within Range	Stable	Stable	Stable
C-reactive Protein (CRP)	0-5 mg/L	Elevated (Baseline)	147	390	114
Serum Lactate	0.5-2.2 mmol/L	Within Range	Stable	5	2
Serum Troponin	0-47 ng/L	Not Done	Normal	72 & 69 (after 3 Hrs)	Stable
Serum Urea	2.5-7.8 mmol/L	Within Range	Stable	27.5	8
Serum Creatinine	59-104 μmol/L	Within Range	Stable	198	120
eGFR	>90ml/min	Within Range	Stable	32	70
Albumin	35-50 g/L	Within Range	Stable	28	30

**Figure 1:**
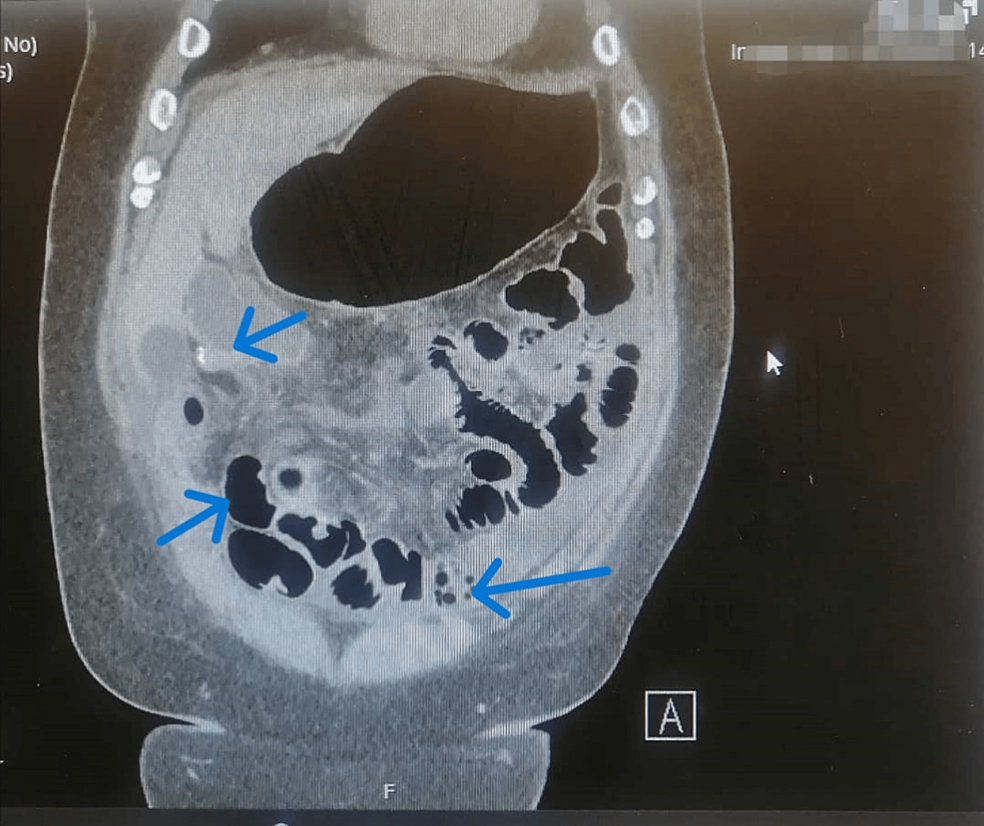
CT of the abdomen and pelvis with oral and IV contrast on POD 5 showing a dilated small intestine with no transitional point. The colon contains gas extending to the rectum, suggesting ileus rather than obstruction, and there is no evidence of a leak. POD: Postoperative day.

A CT abdomen and pelvis with oral and IV contrast (CTAP) was performed, ruling out an anastomotic leak and collection, and indicated only the ileus without obstruction (Figure 1). Ongoing subsequent tachycardia, desaturation, and temperature spikes prompted a septic screen and transfer to the CCU. Despite no evidence of a leak, the patient's condition deteriorated, leading to intubation on POD 7. An ECG was performed in the CCU and showed ST depression in leads V2-V4, and Troponin levels were elevated. Blood parameters indicated a positive Coagulase-negative Staphylococcus from the central venous catheter (CVC) line blood culture, which was sensitive to Meropenem; they also showed deranged renal function and an elevated CRP (Table 1).

**Figure 2:**
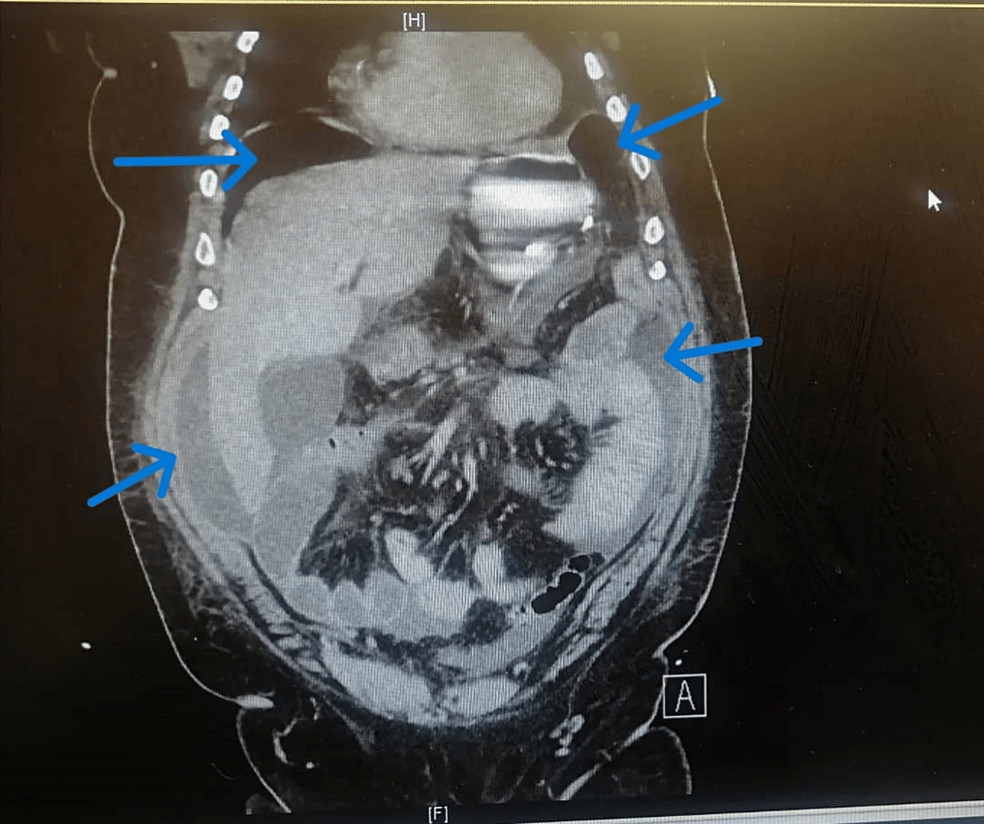
CT of the abdomen and pelvis with oral and IV contrast on POD 7 showing increased pneumoperitoneum and free fluid below both diaphragms, suggesting perforation with features of peritonitis. The ileocolic anastomosis appears unremarkable. POD: Postoperative day.

**Figure 3:**
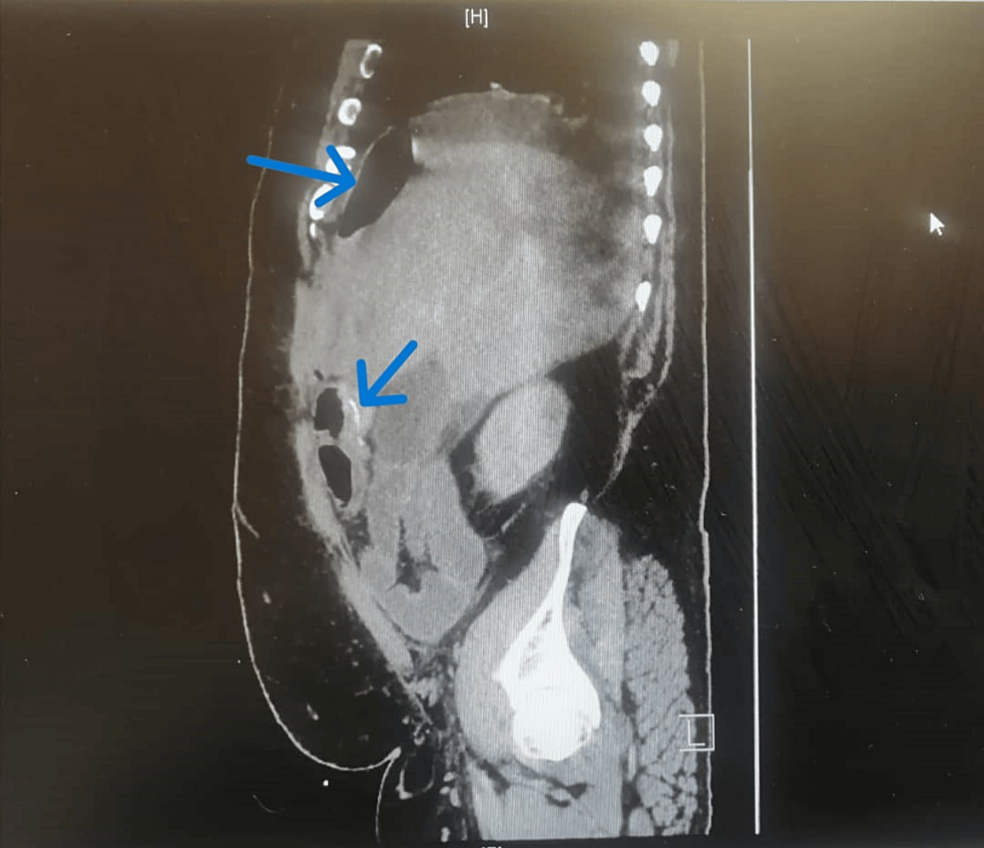
CT of the abdomen and pelvis with oral and IV contrast on POD 7 showing increased pneumoperitoneum, suggesting perforation with features of peritonitis. The ileocolic anastomosis appears unremarkable. POD: Postoperative day.

A repeated CTAP on POD 7 revealed increased pneumoperitoneum and free fluid, suggesting perforation with peritonitis, although the ileocolic anastomosis appeared unremarkable (Figures 2-3). The investigation findings were discussed in the MDT meeting on the same day. This reinforced the absence of a leak but highlighted suspicions of perforation, evolving sepsis, and the potential for systemic infection.

Differential diagnoses

On POD 5, the primary differentials included paralytic ileus and anastomotic leak with intraperitoneal collection, although it was early for an anastomotic leak to occur. The first CTAP with contrast ruled out a leak and confirmed features of the ileus. On POD 6, the patient became tachycardic and tachypneic, experienced temperature spikes, and had left-sided chest pain radiating to the shoulder. This prompted considerations for myocardial infarction (MI), pulmonary embolism (PE), and chest infection, given preoperative risks. An ECG showed ST depression, and elevated troponin levels indicated potential cardiac involvement (Table 1). An urgent chest X-ray revealed left basal atelectasis and consolidation. A cardiology consultation ruled out MI. Subsequently, stat doses of intravenous antibiotics, Bisoprolol, and Glyceryl trinitrate (GTN) spray stabilized the patient. On POD 7, severe abdominal pain, generalized tenderness, tachycardia, tachypnea, fever, and low urine output raised concerns again for an anastomotic leak and PE. The second CTAP on POD 7 confirmed evidence of a leak, while a CT pulmonary angiogram ruled out PE.

Treatment

On POD 7, a detailed discussion was conducted with the patient's family. The critical situation of the patient was explained, and the plan for urgent intervention was discussed, along with the pros and cons of the planned operation. Subsequently, the patient underwent an emergency laparotomy, which revealed fecal peritonitis in all quadrants. Intraoperatively, an anterior defect in the ileocolic anastomosis (approximately 0.5 cm) was identified. Due to a short, friable mesentery with dense inflammatory adhesions, large body habitus, and a substantial layer of adipose tissue, the formation of a stoma was considered unsafe. Therefore, the defect was controlled using a 30Fr Foley catheter with purse-string sutures around the defect, secured to the anterior abdominal wall. Extensive washout and the insertion of four 28Fr drains in the right upper and lower quadrants, left paracolic gutter, and pelvis were performed, followed by mass closure. Intraoperatively, he received two units of Albumin and four units of Fresh Frozen Plasma. Postoperatively, he was admitted to the Critical Care Unit (CCU) and connected to a mechanical ventilator.

Outcome and follow-up

**Figure 4:**
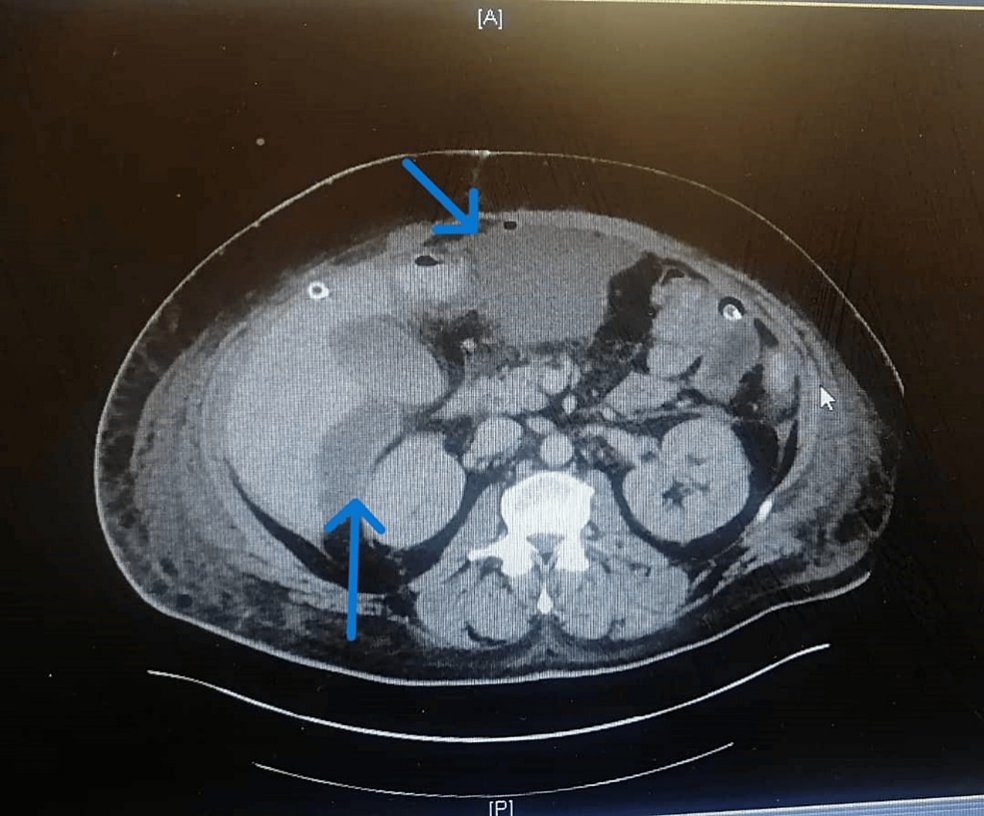
A follow-up CT of the abdomen and pelvis with oral and IV contrast showing sizable undrained collections anteriorly, in the subhepatic region, and at the left upper quadrant.

Over the initial five postoperative days, the patient exhibited stability while on noradrenaline, with improved inflammatory parameters. The presence of an intraperitoneal leak was indicated by feculent fluid drainage via the anastomotic Foley catheter and the right subdiaphragmatic drain. Cessation of inotropes and improved intra-abdominal pressure were observed two days later. A follow-up CTAP revealed sizable undrained collections, leading to CT-guided drainage of 200 ml of pus-stained fluid (Figure 4). By POD 14, due to prolonged intubation, the patient underwent tracheostomy, continued recovery, and his renal function began to improve (Table 1).

On POD 26, the ongoing anastomotic leak was controlled via the initial right sub-diaphragmatic drain. Weaning from mechanical ventilation began, the patient started mobilization with physiotherapy, abdominal drains were removed, and follow-up CT on POD 35 showed only minimal collections. By POD 40, the patient was discharged from the CCU and resumed a normal diet. The Foley catheter was removed on postoperative day 40 after the patient had opened his bowel multiple times. Following the removal of the Foley catheter, the fistula was packed, and it closed spontaneously 4 days later. Then, the patient was discharged home on oral antibiotics and planned for systemic anti-cancer therapy (SACT) after completing the antibiotics course as per the Pathology MDT decision

## Discussion

Postoperative anastomotic leaks following oncological right hemicolectomy pose serious complications associated with increased morbidity and mortality [5]. An international multicenter study reported an overall ileocolic anastomotic leak rate of 7.4%, with 30-day morbidity and mortality rates of 38% and 10.6%, respectively [6]. Early leaks within 30 days often result in peritonitis and high mortality, while late leaks can lead to persistent pelvic abscesses [7]. The risk factors for anastomotic leaks include non-modifiable risks such as age (<60 years (6.4% risk), 60-69 years (5.5% risk), ≥70 years (4.9% risk)), male sex, and certain comorbidities (such as diabetes, pulmonary, renal, and vascular diseases) [3,8]. Modifiable risks involve smoking, alcohol and malnutrition, obesity (BMI >30), anemia (Hb < 11 g/dl), chemotherapy, and immunosuppressant therapy [9-12]. Recent studies have shown that perioperative blood transfusion, emergency surgery, and operative time exceeding three hours are risk factors for anastomotic leaks [13-15]. Anastomotic leaks can be diagnosed radiologically via CT scan with contrast, clinically when there is leakage of gas or bowel content through the wound or drains, endoscopically and intraoperatively [16].

Major anastomotic leakage post-right hemicolectomy correlates with male sex, hypertension, and intraoperative blood transfusion [17]. Early diagnosis is crucial, and symptoms like tachypnea, tachycardia, temperature spikes, oliguria, and mental status changes should prompt further investigation. One critical drawback of anastomotic leakage is the delay in starting adjuvant chemotherapy, especially for stage III colorectal cancer patients. Kim et al. evaluated the effect of anastomotic leakage following colorectal cancer surgery on the timing of receiving and starting adjuvant chemotherapy in 33 patients with a leak versus 776 patients without a leak [18]. In stage III disease, the 5-year local recurrence rates were 59.4% and 9.1% for the leak and non-leak groups, respectively (p < 0.001) [18]. The likelihood of receiving adjuvant therapy for stage III colorectal cancer was lower in the leak group (63% vs. 87%, p = 0.007), and the mean time to the initiation of chemotherapy was longer in the leak group (52 days vs. 37 days), although this was statistically insignificant (p = 0.080) [18]. The insertion of tubes and fecal diverting devices during bowel surgery is common [19]. However, the use of Foley catheters in treating ileocolic anastomotic leaks has not been documented. The routine surgical practice involves breaking down the anastomosis and creating a proximal ileostomy. In challenging cases where stoma creation is unsafe, using a Foley catheter through the anastomotic defect while preserving it can be a viable option. Therefore, surgeons should be aware of intraoperative limitations when performing routine emergency procedures, necessitating a broader repertoire of surgical options for dealing with intraoperative difficulties. This case report underscores the importance of a multidisciplinary approach in managing complex patients and contributing to favorable outcomes.

## Conclusions

Anastomotic leaks are common in colorectal cancer patients, emphasizing the crucial role of early diagnosis in reducing morbidity. In metastatic colorectal cancer patients, a multidisciplinary team approach is a cornerstone for diagnosing, treating, and effectively managing postoperative complications, ensuring a favorable outcome. In situations where creating a stoma may pose safety concerns, inserting a defunctioning tube through the anastomotic defect without disrupting the anastomosis is a secure alternative. It is essential to prepare patients' families for an extended hospital stay, numerous follow-up scans, radiologically guided drainage procedures, and the potential delay in initiating adjuvant chemoradiotherapy.
